# A chromosome-level genome assembly of Chinese quince (*Pseudocydonia sinensis*)

**DOI:** 10.3389/fpls.2024.1368861

**Published:** 2024-05-31

**Authors:** Ying Yang, Jin Feng Liu, Xian Feng Jiang

**Affiliations:** ^1^ College of Agriculture and Biological Science, Dali University, Dali, Yunnan, China; ^2^ Co-Innovation Center for Cangshan Mountain and Erhai Lake Integrated Protection and Green Development of Yunnan Province, Dali University, Dali, Yunnan, China

**Keywords:** comparative genomic analysis, *Chaenomeles sinensis*, Chinese quince, *Pseudocydonia sinensis*, genome

## Abstract

**Introduction:**

*Pseudocydonia sinensis*, also known as Chinese quince, is a perennial shrub or small tree highly valued for its edibility and medicinal properties.

**Method:**

This study presents the first chromosome-level genome assembly of *P. sinensis*, achieved using HiFi sequencing and Hi-C scaffolding technology.

**Results:**

The assembly resulted in a high-quality genome of 576.39 Mb in size. The genome was anchored to 17 pseudo-chromosomes, with a contig N50 of 27.6 Mb and a scaffold N50 of 33.8 Mb. Comprehensive assessment using BUSCO, CEGMA and BWA tools indicates the high completeness and accuracy of the genome assembly. Our analysis identified 116 species-specific genes, 1196 expanded genes and 1109 contracted genes. Additionally, the distribution of 4DTv values suggests that the most recent duplication event occurred before the divergence of *P. sinensis* from both *Chaenomeles pinnatifida* and *Pyrus pyrifolia*.

**Discussion:**

The assembly of this high-quality genome provides a valuable platform for the genetic breeding and cultivation of *P. sinensis*, as well as for the comparison of the genetic complexity of *P. sinensis* with other important crops in the Rosaceae family.

## Introduction

**Figure 1:**
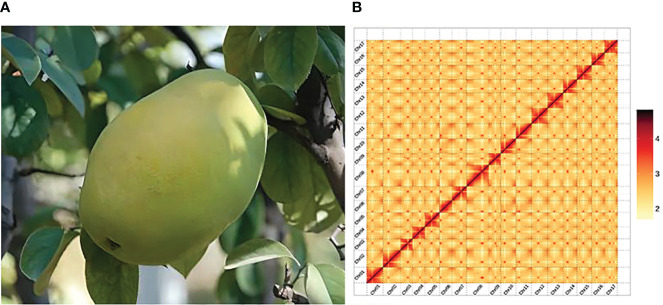
Plant morphology and Hi-C-assisted genome assembly of *P. sinensis*. **(A)** Phenotype of the sequenced *P. sinensis* plant. **(B)** Hi-C interaction heatmap showing 100-kb resolution super scaffolds.

*Pseudocydonia sinensis* (Thouin) C. K. Schneid., also known as Chinese quince, is a shrub or small tree belonging to the genus *Pseudocydonia* in the subfamily Maloideae of Rosaceae (**Figure 1A**). It is the only species in the genus *Pseudocydonia*. This species is native to the central and eastern regions of China, and introduced to numerous countries around the world (Lu et al., 2003). *P. sinensis* blooms from March to April and fruits from June to July. The plant produces pale red flowers, and its fruits are yellow-green, oblong in shape, emit distinct fragrance and have a noticeable tart taste. *P. sinensis* is valued for its ornamental beauty, edibility, and medicinal value. In various regions of China, the fruit is consumed by locals through boiling in water or preserving in sugar. Extracts from the fruit are also widely used in the production of candies, beverages and desserts. Medically, the dried *P. sinensis* fruit is known for its hangover relief and expectorant properties (Grygorieva et al., 2020).

*P. sinensis* exhibits a notably close genetic and morphological relationship with the genus *Chaenomeles* (Koehne, 1890; Sun et al., 2020a), and with the genus *Cydonia* (also named as quince) (Monka et al., 2014). In some taxonomic treatments, it is classified within the genus *Chaenomeles* (as *Chaenomeles sinensis* (Thouin) Koehne). This genus also includes *Chaenomeles cathayensis*, *Chaenomeles japonica*, *Chaenomeles speciosa*, and *Chaenomeles thibetica* (Koehne, 1890). Utilizing fragment sequencing and morphological evidences, various studies delineated *P. sinensis* as distinct from the genus *Chaenomeles*, thereby classifying it as a monotypic genus (Robertson et al., 1991; Campbell et al., 1995; Aldasoro et al., 2005).

Karyotype analysis of *P. sinensis* revealed that the species has a small genome size, with a diploid number of 34 chromosomes, ranging from 1.0 to 1.8 μm in length (Iwatsubo et al., 2022). Despite being widely cultivated as an ornamental and dual-purpose plant, no complete genome of *P. sinensis* has been sequenced to date, posing a significant limitation for the breeding and evolutionary studies of this species. Here, we present the first chromosome-level genome assembly of *P. sinensis*, relying on Hifi sequencing and Hi-C scaffolding technology. Utilizing this high-quality genome assembly, we conducted a comparative genomic analysis between *P. sinensis* and 12 other Rosaceae species, and investigated the genomic structural differences between *Pseudocydonia sinensis*, *Crataegus pinnatifida* and *Pyrus pyrifolia*.

## Materials and methods

### Samples collection and DNA extraction

Samples were collected from an agricultural plantation in Dali, Yunnan province (E 100.191766, N 25.690538), comprising leaves, stems, and fruits of a single *P. sinensis* tree. high-quality genomic DNA was extracted from the sampled leaves using the CTAB method (Porebski et al., 1997). Subsequently, the purity and concentration of DNA were assessed with Nanodrop (Technologies, Wilmington, DE), Qubit 3.0 fluorometer, and electrophoresis on a 1% agarose gel.

### Genome survey of *Pseudocydonia sinensis*

The genome size of *P. sinensis* was estimated using k-mer method (Marçais and Kingsford, 2011) based on Illumina genomic DNA sequencing data. A high-quality Illumina DNA library was constructed and sequenced using Illumina NovaSeq platform with the PE150 layout. This process yielded a total of 70.3 Gb of raw sequencing data. To ensure data reliability, stringent quality filtering was applied to the raw data. Ultimately, 70.2 Gb of high-quality clean reads were obtained for use in genomic exploration and refinement. Quality-filtered reads were subjected to k-mer analysis using Jellyfish 2.0 program (http://www.genome.umd.edu/jellyfish.html).

### DNA and RNA extraction and sequencing

For PacBio HiFi sequencing, high quality genomic DNA was extracted and purified from *P. sinensis* leaves. DNA samples that passed quality checks (main band >30kb) were selected to be randomly fragmented into pieces (15–18kb). The DNA fragments were enriched and purified followed by end repaired. Adapters were ligated to both ends of the nucleic acid fragments, and a library was constructed by removing unsuccessfully ligated fragments with exonuclease. The constructed library was then sequenced on the PacBio Sequel II platform, and the raw data was processed using the CCS program (https://github.com/PacificBiosciences/ccs) to generate HiFi reads.

The Hi-C library was prepared according to Belton et al. (2012) and Shi et al. (2019) with a modification. The extracted genomic DNA was randomly fragmented into pieces and labeled with biotin-14-dCTP. A library was constructed followed by blunt-end repaired, A tailing, adapter ligation, purification and PCR amplification. The Hi-C libraries were quantified and sequenced on the Illumina NovaSeq platform using the PE 150 layout, yielding 65.5 Gb of data. Quality control of Hi-C raw data was performed using the software HiC-Pro v2.8.0 (Belton et al., 2012).

Total RNA Extraction Kit (RNAprep Pure DP441) was used to isolate total RNA from three different tissues (leaf/stem/fruit) of a single *P. sinensis* tree. Eukaryotic mRNA was enriched from the total RNA. Single-stranded cDNA was synthesized using random hexamers as primers with the mRNA as a template, and thus to synthesize double-stranded cDNA, resulting in the final sequencing library. The qualified libraries were pooled and sequenced on the Illumina platform at Novogene Bioinformatics Technology Co., Ltd. (Beijing, China).

### Genome assembly, polishing, and quality evaluation

The size and heterozygosity of the *P. sinensis* genome were estimated using k-mer statistics (Marçais and Kingsford, 2011) (k=17). HiFiasm (https://github.com/chhylp123/hifiasm) (Cheng et al., 2021) in combination with HiFi data provided precise local haplotype information. Contigs were constructed from high-quality HiFi reads derived from the PacBio sequencing dataset. Nextpolish v1.3.1 (Hu et al., 2020) (https://github.com/Nextomics/NextPolish) was applied to rectify errors in the assembled contigs.

Hi-C technology was used to provided long-range interaction information to achieve global phasing of the genome (Burton et al., 2013). The sequenced Hi-C data were assembled at the chromosomal level using Allhic software (https://github.com/tangerzhang/ALLHiC) (Dudchenko et al., 2017). The Juicebox software (Dudchenko et al., 2018) was used to manually correct the chromosome interaction intensity, and finally obtain the genome at the chromosome level. Chromosomal interaction heatmap was used to visualize the interaction matrices of each chromosome (Wolff et al., 2020).

To assess the quality of the *P. sinensis* genome assembly, the completeness of *P. sinensis* genome was assessed using BUSCO v5.2.2 (https://busco.ezlab.org) (Simão et al., 2015) and CEGMA v2.5 (Parra et al., 2007). BWA v0.7.8 (https://github.com/lh3/bwa) (Li and Durbin, 2009) was used to align the Illumina short-read libraries to the assembled genome, calculate the mapping rates, the genome coverage, and the sequencing depth. Mequery (https://github.com/marbl/merqury) was employed to assess the consistency quality values (QV) of the genome assembly.

### Genome annotation

The repeat annotation prediction utilized a combined strategy integrating homology alignment and *de novo* prediction. RepeatMasker software (www.repeatmasker.org) (Tempel, 2012) and its in-house script Repeatproteinmask (http://www.repeatmasker.org/) were employed to detect homologous sequences from the Repbase (http://www.girinst.org/repbase). Tandem repeat sequences were extracted using TRF program (http://tandem.bu.edu/trf/trf.html). And ab initio prediction was conducted by LTR_FINDER (http://tlife.fudan.edu.cn/ltr_finder/) (Xu and Wang, 2007), RepeatScout (http://www.repeatmasker.org/) (Price et al., 2005) and RepeatModeler (http://www.repeatmasker.org/RepeatModeler.html) (Flynn et al., 2020). A custom library in combination with the aforementioned databases was provided to RepeatMasker for the DNA-level repeat identification.

The genome structural annotation incorporated ab initio prediction, homology-based prediction and RNA-Seq assisted prediction. Augustus (v3.2.3) was applied for the gene predication based on ab initio. Sequences of homologous proteins were downloaded from Ensembl/NCBI/others. Protein sequences were aligned to the genome using TblastN (v2.2.26, E-value ≤ 1e-5). To optimize the genome annotation, the RNA-Seq reads from different tissues (leaf/stem/fruit) were aligned to the genome using TopHat (v2.0.11) (Trapnell et al., 2009). The alignment results were then provided to Cufflinks (v2.2.1) for genome-based transcript assembly. Gene functions were assigned using Blastp (with a threshold of E-value ≤ 1e-5). The motifs and domains were annotated using InterProScan (v4.8). We predicted the proteins function by transferring annotations from the closest BLAST hit (E-value <10–5) in the Swissprot database and BLAST hit (E-value <10–5) in the NR database. The tRNAs were predicted using the program tRNAscan-SE (http://lowelab.ucsc.edu/tRNAscan-SE/). Other ncRNAs, including miRNAs, snRNAs were identified by searching against the Rfam database with default parameters using the infernal software (http://infernal.janelia.org/).

### Genomic evolution history analysis

Genomic sequences of 12 other Rosaceae species (*Crataegus pinnatifida, Fragaria vesca, Gillenia trifoliate, Malus sieversii, Malus dometic, Potentilla anserine, Prunus armeniaca, Prunus avium, Pyrus pyrifolia, Rosa chinensis, Rubus idaeus, Rubus occidentalis*) were gathered from various databases (**Supplementary Table 1**). AGAT (v1.0.0) (https://github.com/NBISweden/AGAT) was utilized to standardize the genome sequences for all species by retaining the protein-coding genes and the longest transcripts. The Coding sequences (CDS) and protein encoding sequences (PES) were filtered out with TransDecoder (https://github.com/TransDecoder/TransDecoder). Orthofinder v2.5.4 (https://github.com/davidemms/OrthoFinder) (Emms and Kelly, 2019) was applied to cluster the gene families. ParaAT (v2.0) (Zhang et al., 2012) was utilized to perform the multiple sequence alignment based on orthologous single-copy genes. The aligned sequences were merged into supergenes, and the non-conserved regions were trimmed using Trimal (v1.2) (Capella-Gutiérrez et al., 2009). IQ-TREE (Nguyen et al., 2014)was utilized to construct the maximum likelihood (ML) phylogenetic tree based on 165 orthologous single-copy genes (bootstrap =1000). *A. thaliana* was used as the outgroup, and the phylogenetic tree was visualized using the ggplot2 package in R.

The time calibration was conducted based on available TimeTree (http://timetree.org/) fossil records. The fossil time calibration was conducted based on the root nodes of *A.thaliana* and Rosaceae species, as well as the root nodes of *C. pinnatifida* and *P. pyrifolia*, resulting in a rooted tree with fossil time calibrations. The divergence times between species were calculated using the MCMCTree in the PAML software (Yang, 2007). FigTree (http://tree.bio.ed.ac.uk/) was used to visualize the phylogenetic tree with divergence times. The calculation of contraction and expansion gene families for each lineage was conducted using CAFE5 software (https://github1s.com/hahnlab/CAFE) (De Bie et al., 2006). Contraction and expansion genes families of *P. sinensis* were further analyzed for the GO enrichment analysis using ClusterProfile software (Yu et al., 2012).

The genomes of *P. sinensis*, *C. pinnatifida* and *P. pyrifolia* were selected for the analysis of whole-genome duplication (WGD) events and selective pressure analysis. The WGD event was determined by the fourfold synonymous third-codon transversion (4DTv) values (Yang and Nielsen, 2000). JCVI (https://github.com/tanghaibao/jcvi/wiki/MCscan-(Python-version)) was used to identify the homologs among three species. ParaAT (https://ngdc.cncb.ac.cn/tools/paraat) was used to perform protein-coding DNA alignments for these homologs. The non-synonymous/synonymous substitution (Ka/Ks) values were calculated to assess the selective pressure using the YN00 program of the PAML software with default parameters (Yang, 2007). The Ks values and 4DTv were visualized using the ggplot2 package in R. MCScanX (Wang et al., 2012) was used to identify syntenic regions and generate a synteny plot between the three species.

## Results

### Genome sequencing and assembly

Illumina sequencing yielded 70.2 Gb of clean reads with a coverage depth of 119.25X. Genome survey analysis using kmer (k=17) indicated a primary peak around depth=36, and the estimated genome size calculated by the formula Kmer-number/depth is approximately 679.04 Mb, with a corrected genome size of 664.59 Mb. The genome heterozygosity rate is 0.62%, and the proportion of repetitive sequences is 55.05% (**Supplementary Table 2**, **Supplementary Figure 1**).

A high-quality DNA sample was used to construct a SMRTbell library, which was sequenced on the PacBio Sequel II platform with a coverage depth of 35.78X, yielding a total of 21 Gb of HiFi reads after a series of processing steps. The HiFi reads comprised 2,456,334 reads, with an average read length of 8,660 bp, a N50 read length of 12,347 bp, and the longest read length being 49,180 bp (**Supplementary Table 3**, **Supplementary Figure 2**). Hifiasm resulting in a primary assembly comprising 301 contigs, with a total contig length of 576.39 Mbp and a contig N50 length of 27.6 Mbp (**Supplementary Table 4**).

The Hi-C library sequencing yielded a total of 67.07 Gb of raw sequencing data, 66.66 Gb of clean Hi-C data were obtained after filtering. The *P. sinensis* genome was assembled to chromosome-level resolution with the aid of Hi-C data. The resulting chromosomal genome assembly yielded a total contig length of 576,387,120 bp and a contig N50 size of 27,604,817 bp; the total scaffold length amounted to 576,390,020 bp, with a scaffold N50 size of 33,874,332bp. The genome anchoring rate was 97.62% (**Supplementary Tables 5**, **6**). The chromosomal interaction heatmap displayed 17 clear chromosomal clusters, with interactions within individual chromosomes being markedly higher than that between chromosomes (**Figure 1B**).

The BUSCO assessment yielded a completeness score of 99.00%. The CEGMA analysis utilized a core gene set consisting of 248 conserved genes obtained from six eukaryotic model organisms. In *P. sinensis*, 241 out of 248 Core Eukaryotic Genes (CEGs) genes were assembled, achieving a final ratio of 97.18%. BWA software aligned short-read library data with the assembled genomic sequence of *P. sinensis*, achieving an approximate read alignment rate of 99.35% and a genome coverage of about 99.97% (**Supplementary Table 7**, **Supplementary Figure 3**). The QV value derived from the mergury-mash module of Merqury software was 48.8606. By calculating the GC content and average depth of the assembled genome sequence, it is proved that there is no GC bias and possible contamination in the analyzed sequencing data (**Supplementary Figure 4**). Overall, the chromosomal assembly and genome anchoring rates were favorable, indicating that the genome assembly was highly satisfactory. The overall statistics for the *P. sinensis* genome are listed below (**Table 1**).

**Table 1:** Summary statistics for the *P. sinensis* genome.

Features	value
Estimated genome size (Mb)	664.59
Total contigs length of assembly(Mb)	576.39
Number of contigs	301
Contig N50(Mb)	27.6
Largest contig (Mb)	38.3
Number of scaffolds	272
Scaffold N50	33.8
Largest scaffold	48.7
Chromosome length(Mb)	562.7
GC content (%)	36.90
Heterozygosity (%)	0.62%
Number of coding genes	37,779

### Genome annotation

**Figure 2:**
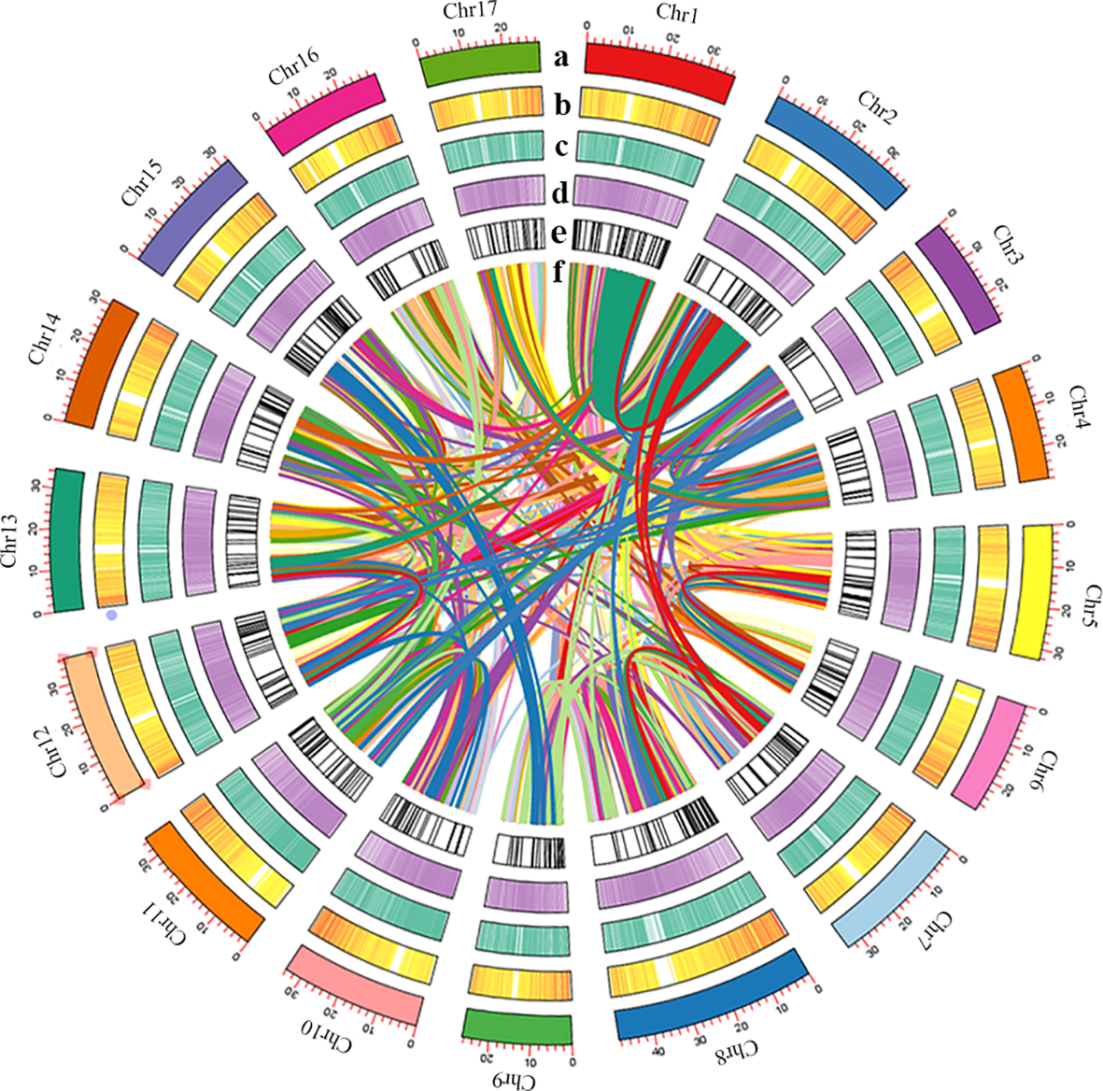
**(A)** Length of each pseudomolecule, **(B)** density of gene, **(C)** heatmap of GC content, **(D)** density of transposon, **(E)** non-coding RNA, and **(F)** events shown by syntenic relationships.

313,631,790 bp tandem repeat sequences were obtained, makes up 54.41% of the *P. sinensis* genome (**Supplementary Table 8**). For a total of 280,849,280 bp long terminal repeats (LTRs) were get, which is the most abundant class for the repeat sequences, comprising 48.73% of the genome (**Supplementary Table 9**). A total of 37,779 protein-coding genes were predicted, of which 22,301 could be structurally predicted by all three methods (*de novo* prediction, homology prediction, and transcriptome-assisted prediction). Within the coding genes, the average lengths of transcripts and CDS are 3,116.70 bp and 1,189.34 bp, respectively. The average lengths of exons and introns are 230.81 bp and 464.10 bp, respectively, with an average of 5.15 exons per gene (**Supplementary Table 10**). 37,398 out of the 37,779 protein-coding genes could be annotated, while 381 remained unannotated. The probability of predicting gene function was 98.99% (**Supplementary Table 11**). As for the non-coding genes, 637 miRNAs, 1,408 tRNAs, 599 snRNA and 5,572 rRNA were identified from the *P. sinensis* genome (**Supplementary Table 12**). An overview of the genome assembly and annotation is shown in **Figure 2**.

### Gene family and evolution analysis

**Figure 3:**
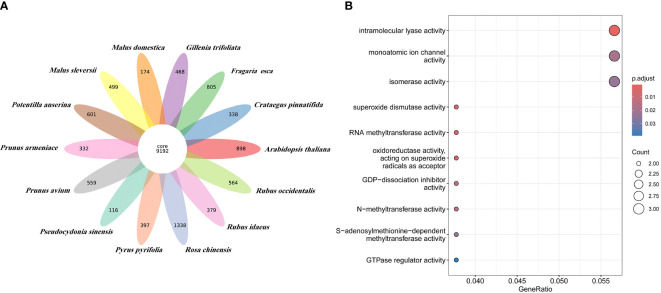
Orthologous clustering of *P. sinensis* genome. **(A)** Venn diagram of the number of shared gene families between *P. sinensis* and other 13 species; **(B)** GO enrichment shows the function of the species-specific genes of *P. sinensis*.

Orthologous clustering results identified a total of 36,911 orthologous gene families across these 14 species. 9,192 gene families were identified to be shared by all species (see **Figure 3A**). 116 species specific gene families of *P. sinensis* were identified for the 13 species closely related to *P. sinensis* (**Supplementary Table 13**). GO enrichment suggests that these species-specific genes are primarily involved in the regulation of redox reactions, cleavage reactions, ion channel regulation, signal transduction, and methylation modification (**Figure 3B**).

**Figure 4:**
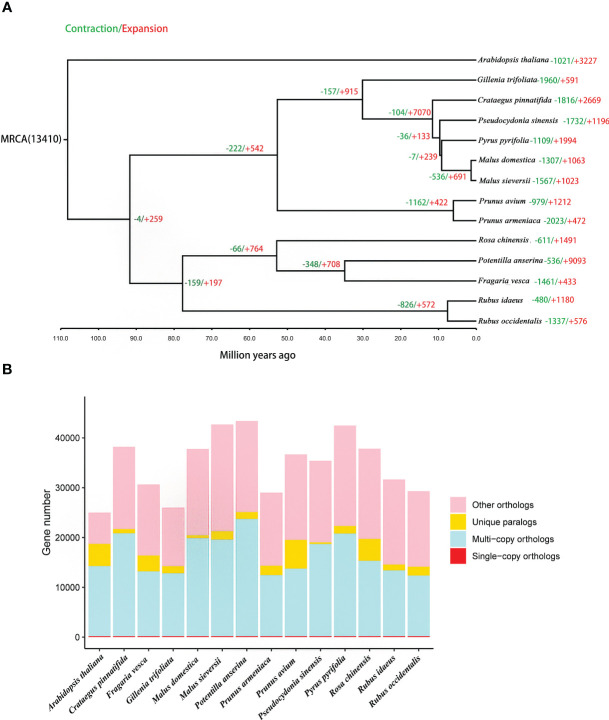
Gene family and phylogenetic tree analyses of *P. sinensis* and other related plant species. **(A)** phylogenetic tree based on shared single-copy gene families and gene family expansions and contractions among *P. sinensis* and 13 other species, **(B)** Gene family clustering in *P. sinensis* and 13 other plant genomes.

A total of 165 orthologous single-copy genes were identified in the 14 species, (**Figure 4B**; **Supplementary Table 14**). The phylogenetic results indicate that *P. sinensis* share a common ancestor with *P. pyrifolia, M. sieversii* and *M. domestica*. *P. sinensis* and *P. pyrifolia* diverged from their common ancestor around 11.6 Mya. Meanwhile, *P. sinensis* and *C. pinnatifida* diverged from a shared ancestor around 30 Mya (**Figure 4A**; **Supplementary Figure 5**).

**Figure 5:**
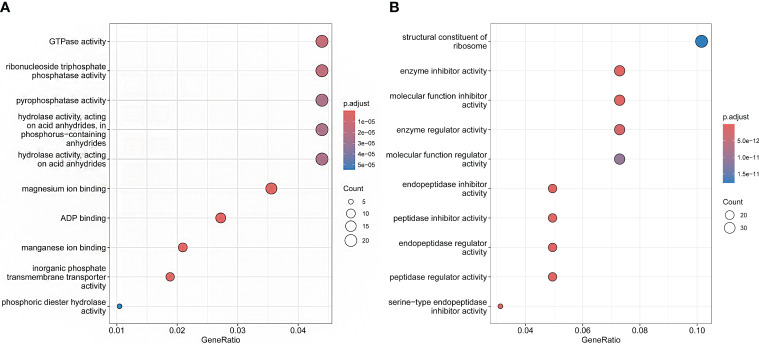
GO enrichment shows the functions of the expansion and contraction genes of *P. sinensis*. **(A)** the functions of contraction genes, **(B)** the functions of expansion genes.

In the analysis of expansion and contraction gene families, 1196 significantly expanded and 1732 significantly contracted gene families were detected for *P. sinensis* (**Figure 4A**). According to the GO enrichment results, the expanded gene families are primarily associated with protein synthesis, regulation, and degradation. In contrast, the contracted gene families are related to nucleotide metabolism and cellular energy metabolism (**Figure 5**).

**Figure 6:**
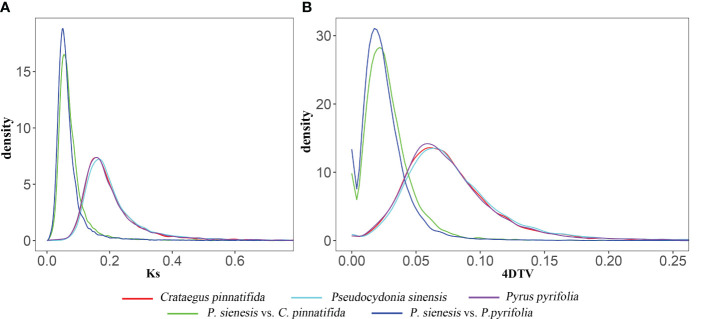
**(A)** Ks and **(B)** 4DTv values of of *P. sinensis*, *C. pinnatifida* and *P. pyrifolia*, as well as *P. sinensis-C. pinnatifida* and *P. sinensis-P. pyrifolia* comparisons.

The Ks and 4DTv values exhibited same trend (**Figure 6A**). The 4DTv values for *P. sinensis*, *C. pinnatifida*, and *P. pyrifolia* indicated that *C. pinnatifida*, *P. pyrifolia* and *P. sinensis* showed a single peak at 0.07, suggesting that *P. sinensis* has undergone only one recent whole genome duplication (WGD) event in its evolutionary history. The most recent duplication event occurred before the divergence of *P. sinensis* from both *C. pinnatifida* and *P. pyrifolia*. Both *P. sinensis*-*C. pinnatifida* and *P. sinensis*-*P. pyrifolia* comparisons revealed a similar peak, with their 4DTv value distributions being roughly equivalent, indicating that these species experienced similar genome duplication events at close evolutionary points (see **Figure 6B**).

**Figure 7:**
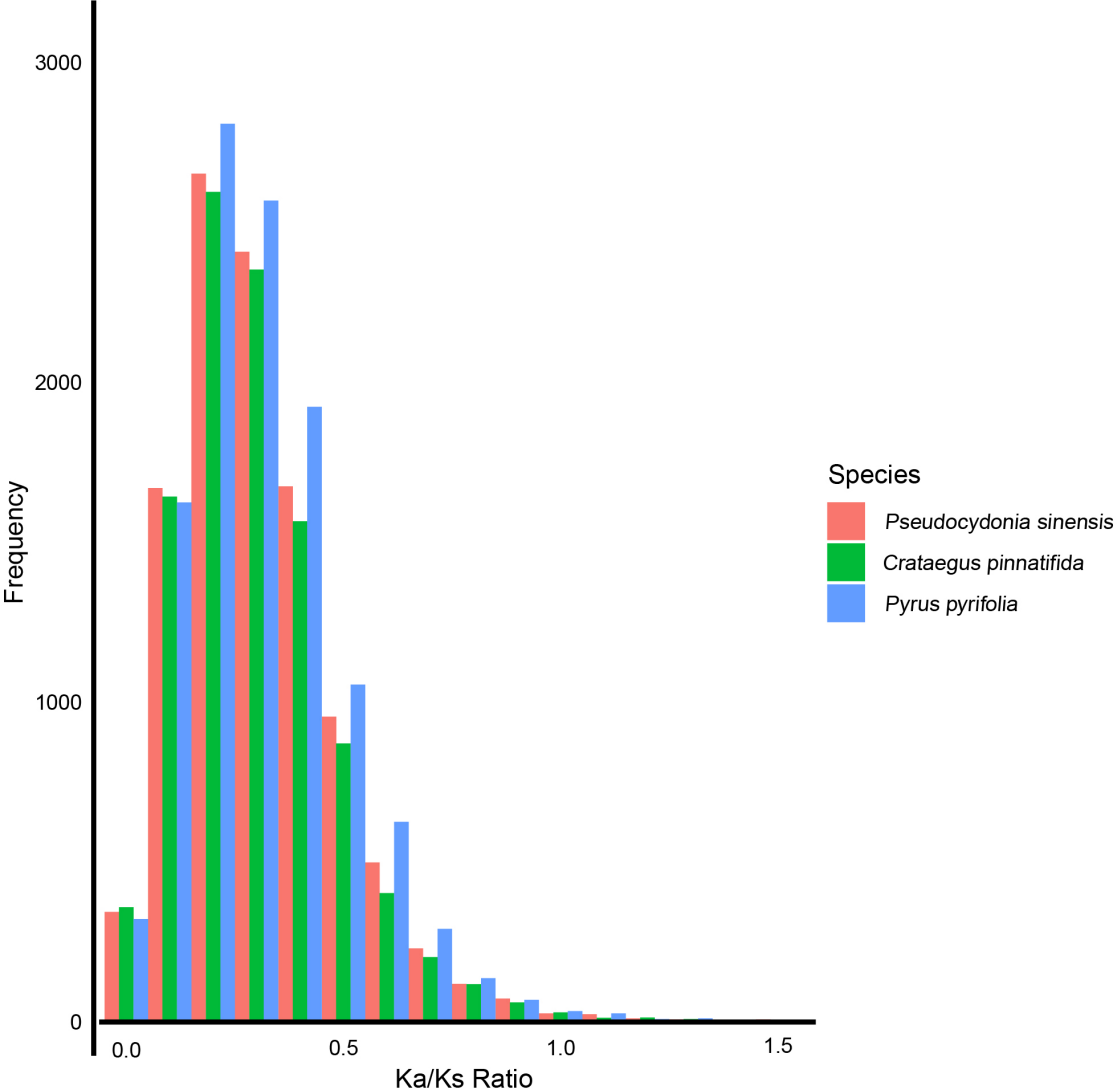
Ka/Ks values of *P. sinensis*, *C. pinnatifida* and *P. pyrifolia*.

Based on Ka/Ks analysis, the selective pressures experienced by *P. sinensis*, *C. pinnatifida*, and *P. pyrifolia* were estimated. We found that most Ka/Ks values of *P. sinensis*, *C. pinnatifida*, and *P. pyrifolia* are less than 1.0. 27 genes of *P. sinensi* were identified positively selected (Ka/Ks>1). When the Ka/Ks values are between 0.2 and 1.1, *P. sinensis* has fewer genes than *P. pyrifolia* and more genes than *C. pinnatifida* (**Figure 7**).

### Genomic synteny analysis

**Figure 8:**
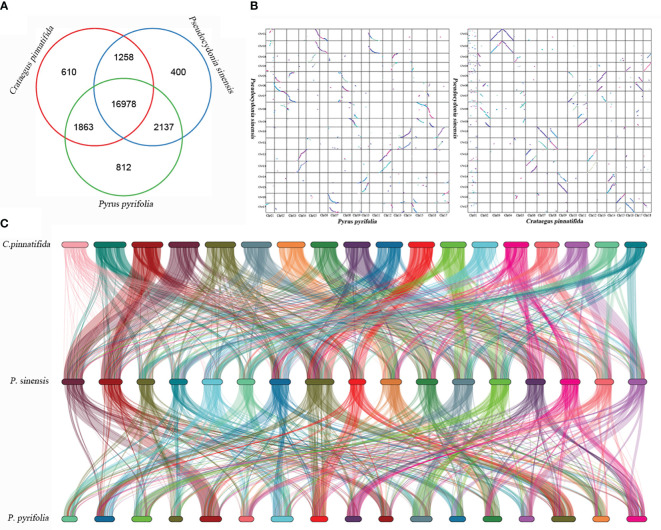
Genomic synteny analysis of *P. sinensis*, *C. pinnatifida* and *P. pyrifolia.* **(A)** Venn diagram of the number of shared gene families within *P. sinensis*, *C. pinnatifida* and *P. pyrifolia*. **(B)** a Dot plots for syntenic genes between *P. sinensis* and *C. pinnatifida*, as well as *P. sinensis* and *P. pyrifolia*. **(C)** Subgenome classification and synteny analysis of *P. sinensis*, *C. pinnatifida* and *P. pyrifolia.*.

*P. sinensis*, *C. pinnatifida* and *P. pyrifolia* share 16,978 similar genes in total (**Figure 8A**). Homologous genes between *C. pinnatifida* and *P. sinensis*, as well as *P. sinensis* and *P. pyrifolia*, were identified based on genomic collinearity analysis. A total of 56,547 collinear genes between *C. pinnatifida* and *P. sinensis* were identified, accounting for 72.17% of the total gene count (78,350), and 58,461 collinear genes between *P. sinensis* and *P. pyrifolia*, comprising 70.73% of the total gene count (82,655). Additionally, there was a significant amount of chromosomal rearrangement events among them, such as translocations (**Figure 8**).

## Discussion

*P. sinensis* serves not only as an ornamental crop, but also valued for its medicinal and edible properties, making it of substantial economic value. However, genetic breeding process in this plant has been relatively low, primarily due to our limited understanding of its genomic background. The sequencing and assembly of present genome provide valuable insights to improve the genetic breeding and cultivation of *P. sinensis* for optimal utilization.

The estimated genome sizes of *P. sinensis* determined by k-mer analysis was 664.59 Mb. The chromosome-level genome of *P. sinensis* assembled with its 576.39 Mb sequence, with a contig N50 size of 27.6 Mb. Hi-C library sequencing generated a total of 67.07 Gb of raw sequencing data, from which 66.66 Gb of clean Hi-C data were obtained. The assembly resulted in a total contig length of 576,387,120 bp and a contig N50 size of 27,604,817 bp; the total scaffold length amounted to 576,390,020bp, with a scaffold N50 size of 33,874,332bp. The genome anchoring rate was 97.62%. The results of chromosome heat map showed 17 distinct chromosome sets, with significantly stronger interaction intensity within chromosomes compared to between chromosomes. The BUSCO assessment indicated a completeness assembly score of 99.00%. the BWA software aligned short-read library data with the assembled genomic sequence, achieving an approximate read alignment rate of 99.35% and a genome coverage of about 99.97%. The comprehensive assessment results indicate that the genome assembly is highly complete and accurate.

The k-mer analysis showed that the heterozygosity rate of the *P. sinensis* genome was 0.62%, which is higher than that in the genomes of peach (0.31%) (Lian et al., 2022), loquat (0.31%) (Wang, 2021), and lower than that in the genomes of pear (0.89%) (Gao et al., 2021), *P. mume* (0.75%) (Zheng et al., 2022), and *Chaenomeles speciosa* (2.1%) (He et al., 2023). The genome size of *P. sinensis* was smaller comparable to that of *Chaenomeles speciosa* (632.3 Mb) (He et al., 2023), apple (652–668 Mb) (Zhang et al., 2019; Sun et al., 2020b), hawthorn (779.24 Mb) (Zhang et al., 2022), loquat (733.32 Mb) (Wang, 2021), but larger than that of pear (496.9–541.34 Mb) (Dong et al., 2020; Gao et al., 2021). We postulated that the smaller genome size might be due to internal standard differences, experimental errors, or other variations among studies.

116 species-specific genes were identified within the *P. sinensis* through orthologous clustering analysis. GO enrichment suggests that these species-unique genes are primarily involved in the regulation of redox reactions, cleavage reactions, ion channel regulation, signal transduction, and methylation modification. 1196 significantly expanded and 1732 significantly contracted gene families were detected in the *P. sinensis* genome. The expanded gene families are primarily associated with protein synthesis, regulation, and degradation. In contrast, the contracted gene families are associated with nucleotide metabolism and cellular energy metabolism. These expansion and contraction play crucial roles in the functional diversification of genes in Rosaceae plants. Previous studies have reported the involvement of expanded gene families in the biosynthetic pathways of plant natural products in the hawthorn genome (Zhang et al., 2022). In the loquat genome, expanded gene families were discovered to be associated with monoterpenoid biosynthesis as well as starch and sucrose metabolism (Wang, 2021).

According to the phylogenetic analysis in this study, *P. sinensis* is found to be closely related to pear and hawthorn (branch support value=100). He et al. (2023) suggest that *Chaenomeles speciose* is more closely related to apple. Due to the unavailability of the whole genome information of *Chaenomeles speciose* at present, it is challenging to conduct a comprehensive comparison of these two species. Nevertheless, considering the available information, it can be inferred that there may not be a strong relationship between the genus *Pseudocydonia* and genus *Chaenomeles*.

WGD events occurred in the genome of *P. sinensis*. According to the 4DTv values, the most recent duplication event occurred before the divergence of *P. sinensis* from both *C. pinnatifida* and *P. pyrifolia*. Consistent with expectations, the distribution of Ks values showed similar trends to that of the 4DTv results. The distribution of Ka/Ks ratios indicates strong negative purifying selection for most genes of *P. sinensis* genome (Ka/Ks<1), while 28 genes were identified as positively. When comparing the syntenic patterns of *P. sinensis* with *C. pinnatifida* and *P. pyrifolia* (**Figure 8C**). Collinearity analysis revealed a large number of homologous gene blocks between each pair of species. We found numerous chromosomal rearrangement and translocation among them, with more chromosomal rearrangement events found between hawthorn and *P. sinensis* than between *P. sinensis* and pear. In the Rosaceae family, conserved syntenic relationships were frequently found between species in the same genus (*Rubus rugosa* and *Rubus chinensis*) (Chen et al., 2021)). However, when comparing with species of other genus in Rosaceae, chromosomal rearrangement and translocation are commonly found, such as between rosa, peach and strawberry (Chen et al., 2021), as well as between *Chaenomeles speciose*, pear and apple (He et al., 2023). These results suggest that the Rosaceae family exhibits conserved synteny within the genus but shows significant genetic variation among genera.

## Data availability statement

The datasets presented in this study can be found in online repositories. The names of the repository/repositories and accession number(s) can be found in the article/**Supplementary Material**.

## Author contributions

YY: Methodology, Software, Writing – original draft. JFL: Data curation, Supervision, Writing – original draft. XFJ: Data curation, Supervision, Writing – review & editing.
