# Supplementary material for: A chromosome-level genome assembly of Chinese quince (Pseudocydonia sinensis)
Source: Front Plant Sci. 2024 May 31;15:1368861. doi: 10.3389/fpls.2024.1368861 (PMC11180997; doi:10.3389/fpls.2024.1368861)
Supplement: Supplementary file 1 [file DataSheet_1.docx]

**Table 1**. accession number of the genome data presented in the study

| species | BioSample | BioProject |
| --- | --- | --- |
| Pseudocydonia sinensis | SAMN39110016 | PRJNA1056826 |
| Malus dometica | SAMN05603590 | [PRJNA339703](https://www.ncbi.nlm.nih.gov/bioproject/PRJNA339703/) |
| Arabidopsis thaliana | SAMN03081427 | [PRJNA10719](https://www.ncbi.nlm.nih.gov/bioproject/PRJNA10719/) |
| Prunus avium | SAMN13640536 |  |
| Rosa chinensis | SAMN07737764 | [PRJNA413292](https://www.ncbi.nlm.nih.gov/bioproject/PRJNA413292/) |
| Crataegus pinnatifida | SAMN27378400 | PRJNA823924 |
| Fragaria vesca |  | PRJNA871257 |
| Gillenia trifoliata | SAMN16480607 | PRJNA669900 |
| Malus sieversii |  | PRJNA591623 |
| Pyrus pyrifolia | SAMD00260485 | [PRJDB10856](https://www.ncbi.nlm.nih.gov/bioproject/PRJDB10856/) |
| Potentilla anserina |  | PRJNA640225 |
| Rubus occidentalis |  | PRJNA430858 |
| Rubus idaeus |  | PRJNA869453 |
| Prunus armeniaca | CNP0000755 (CNSA https://db.cngb.org/cnsa/) | |

**Table 2.** Statistical analysis of Illumina genome survey based on Kmer=17

| Kmer | Depth | Number of kmer | Genome size(M) | Revised Genome_size(M) | Heterozygous rate(%) | Repeat rate(%) |
| --- | --- | --- | --- | --- | --- | --- |
| 17 | 36 | 24,445,343,316 | 679.04 | 664.59 | 0.62 | 55.05 |

**Table 3**. quality assessment results of Pacbio(ccs) data

| Read_type | Read_base | Read_Number | Read_length(max) | Read_length(mean) | Read_length(N50) |
| --- | --- | --- | --- | --- | --- |
| HiFi reads | 21,273,426,545 | 2,456,334 | 49,180 | 8,660 | 12,347 |

**Table4.** statistics of the de novo genome assembly based on hifisam program

| Title | Total_length | Total  number | Average  length | Max  length | Min  length | N50  length | N50  number | N90  length | N90  number |
| --- | --- | --- | --- | --- | --- | --- | --- | --- | --- |
| Contig | 576,387,120 | 301 | 1,914,907 | 38,315,748 | 8,082 | 27,604,817 | 10 | 6,901,936 | 25 |
| Scaffold | 576,387,120 | 301 | 1,914,907 | 38,315,748 | 8,082 | 27,604,817 | 10 | 6,901,936 | 25 |

**Table 5.** Hi-C statistics for the assisted assembly of genome

| Sample ID | Contig length | Scaffold length | Contig number | Scaffold number |
| --- | --- | --- | --- | --- |
| Total | 576,387,120 | 576,390,020 | 301 | 272 |
| Max | 38,315,748 | 48,660,268 | - | - |
| Number>=2000 | - | - | 301 | 272 |
| N50 | 27,604,817 | 33,874,332 | 10 | 8 |
| N60 | 19,860,400 | 32,156,704 | 12 | 10 |
| N70 | 16,963,928 | 29,651,210 | 15 | 12 |
| N80 | 14,334,087 | 28,115,348 | 19 | 14 |
| N90 | 6,901,936 | 27,604,817 | 25 | 16 |

**Table 6.** clustering scaffold number, chromosome sequence length and genome mounting rate for the Hi-C assisted assembly of single chromosome

| Sequeues ID | Cluster Number | Sequence Length |
| --- | --- | --- |
| Chr01 | 3 | 36,695,035 |
| Chr02 | 1 | 38,315,748 |
| Chr03 | 1 | 27,604,817 |
| Chr04 | 1 | 28,115,348 |
| Chr05 | 2 | 32,654,194 |
| Chr06 | 2 | 27,717,457 |
| Chr07 | 1 | 33,874,332 |
| Chr08 | 5 | 48,660,268 |
| Chr09 | 4 | 26,519,663 |
| Chr10 | 1 | 34,020,137 |
| Chr11 | 2 | 35,003,224 |
| Chr12 | 2 | 36,305,265 |
| Chr13 | 4 | 34,775,811 |
| Chr14 | 6 | 31,660,592 |
| Chr15 | 3 | 32,156,704 |
| Chr16 | 3 | 29,651,210 |
| Chr17 | 5 | 28,963,724 |
| Class | Scaffold Number | Total Length |
| place | 17 | 562,693,529 |
| unplace | 255 | 13,696,491 |
| total | 272 | 576,390,020 |
| genome anchoring rate | 97.62% | |

**Table7.** Genomic evaluation based on the reads alignment, CEGs and BUSCOs

| Type 1 | Complete BUSCOs(C) | Complete and single-copy BUSCOs(S) | Complete Duplicated BUSCOs(D) | Fragmented BUSCOs(F) | Missing BUSCOs(M) | Total BUSCO groups searched |
| --- | --- | --- | --- | --- | --- | --- |
| BUSCO assessment | 99.00% | 63.50% | 35.50% | 0.60% | 0.40% | 1614 |
| Type 2 | Number of 248 highly conserved CEGs present in assembly | Percentage of 248 highly conserved CEGs present(%) | \ | \ | \ | \ |
| Core gene integrity assessment | 241 | 97.18% | \ | \ | \ | \ |
| Type 3 | Mapping rate (%) | \ | \ | \ | \ | \ |
| Reads alignment analysis | 99.35% | \ | \ | \ | \ | \ |

**Table 8.** Results of tandem repeats predicted using TRF, Repeatmasker and Proteinmask. The overall result is the non-redundant result of the results obtained by the above methods after the overlap between them is removed.

| Type | Repeat Size(bp) | % of genome |
| --- | --- | --- |
| TRF | 41,550,708 | 7.21 |
| Repeatmasker | 304,264,817 | 52.79 |
| Proteinmask | 65,002,233 | 11.28 |
| Total | 313,631,790 | 54.41 |

**Table 9.** Statistics of repeat sequence classification. **Denovo+Repbase: a** library built with RepeatModeler, RepeatScout and LTR_FINDER software combined with RepBase nucleic acid library, and annotated by RepeatMasker software. **TE Proteins:** The annotation result obtained by RepeatProteinMask software based on RepBase protein library. **Combined TEs:** integrating results after eliminating redundancy.of the two methods.

|  | Denovo+Repbase | | TE Proteins | | Combined TEs | |
| --- | --- | --- | --- | --- | --- | --- |
|  | Length(bp) | % in Genome | Length(bp) | % in Genome | Length(bp) | % in Genome |
| DNA | 19,058,212 | 3.31 | 2,179,614 | 0.38 | 19,639,649 | 3.41 |
| LINE（long interspersed nuclear elements） | 2,901,253 | 0.5 | 1,137,595 | 0.2 | 3,489,705 | 0.61 |
| SINE（short interspersed nuclear elements） | 125,853 | 0.02 | 0 | 0 | 125,853 | 0.02 |
| LTR (long terminal repeats) | 276,694,144 | 48 | 61,685,717 | 10.7 | 280,849,280 | 48.73 |
| Unknown | 8,986,944 | 1.56 | 0 | 0 | 8,986,944 | 1.56 |
| Total | 304,264,817 | 52.79 | 65,002,233 | 11.28 | 307,389,203 | 53.33 |

**Table 10** Statistical results of gene structure prediction

|  | Gene set | Number | Average transcript length(bp) | Average CDS length(bp) | Average exons per gene | Average exon length(bp) | Average intron length |
| --- | --- | --- | --- | --- | --- | --- | --- |
| De novo | Augustus | 39,525 | 2,371.13 | 1,108.39 | 4.58 | 242.21 | 353.1 |
|  | GlimmerHMM | 60,635 | 5,578.32 | 690.3 | 2.92 | 236.63 | 2,549.48 |
|  | SNAP | 44,751 | 3,403.54 | 678.59 | 4 | 169.84 | 909.68 |
|  | Geneid | 61,922 | 4,038.28 | 819.69 | 4.16 | 196.81 | 1,016.97 |
|  | Genscan | 39,756 | 8,397.91 | 1,267.97 | 6.19 | 204.77 | 1,373.17 |
| Homolog | Pavi | 31,068 | 2,931.22 | 1,196.87 | 5.09 | 235.3 | 424.39 |
|  | Parm | 34,240 | 2,595.18 | 1,137.39 | 4.81 | 236.65 | 383 |
|  | Rchi | 31,634 | 2,687.29 | 1,186.01 | 5.03 | 235.96 | 372.86 |
|  | Mdom | 35,100 | 3,202.85 | 1,101.62 | 4.94 | 223.02 | 533.36 |
|  | Ppyr | 34,612 | 2,672.76 | 1,153.36 | 4.93 | 234.02 | 386.77 |
|  | Mbac | 41,764 | 2,301.51 | 974.37 | 4.26 | 228.53 | 406.64 |
| RNAseq | PASA | 15,371 | 2,148.48 | 1,035.90 | 4.01 | 258.54 | 370.03 |
|  | Transcripts | 42,576 | 5,472.34 | 2,281.49 | 6.86 | 332.72 | 544.79 |
| EVM | | 43,429 | 2,872.62 | 1,095.24 | 4.76 | 230.12 | 472.77 |
| Pasa-update* | | 43,410 | 2,818.13 | 1,092.00 | 4.72 | 231.42 | 464.19 |
| Final set* | | 37,779 | 3,116.70 | 1,189.34 | 5.15 | 230.81 | 464.1 |

* Contains the UTR region.

**Table 11** Statistical results of gene function annotation

|  | Number | Percent(%) |
| --- | --- | --- |
| Total | 37,779 | - |
| Swissprot | 28,705 | 75.98 |
| Nr | 37,229 | 98.54 |
| KEGG | 28,372 | 75.1 |
| InterPro | 35,387 | 93.67 |
| GO | 21,808 | 57.73 |
| Pfam | 27,654 | 73.2 |
| Annotated | 37,398 | 98.99 |
| Unannotated | 381 | 1.01 |

**Table 12** Statistical results of non-coding RNA

|  | Type | Copy number | Average length(bp) | Total length(bp) | % of genome |
| --- | --- | --- | --- | --- | --- |
| miRNA | | 637 | 156.2 | 99,499 | 0.017262 |
| tRNA | | 1,408 | 75.53 | 106,342 | 0.01845 |
| rRNA | rRNA | 5,572 | 388.09 | 2,162,460 | 0.38 |
|  | 18S | 859 | 1,726.88 | 1,483,390 | 0.26 |
|  | 28S | 3,240 | 144.29 | 467,503 | 0.081109 |
|  | 5.8S | 821 | 161.96 | 132,967 | 0.023069 |
|  | 5S | 652 | 120.55 | 78,600 | 0.013637 |
| snRNA | snRNA | 599 | 117.58 | 70,429 | 0.012219 |
|  | CD-box | 335 | 105.8 | 35,444 | 0.006149 |
|  | HACA-box | 66 | 128.86 | 8,505 | 0.001476 |
|  | splicing | 194 | 133.44 | 25,887 | 0.004491 |
|  | scaRNA | 4 | 148.25 | 593 | 0.000103 |
|  | Unknown | 0 | 0 | 0 | 0 |

**Table 13**. Results of gene family clustering for the 14 species

| species | Multi-copy orthologs | Other orthologs | Single-copy orthologs | Unique paralogs |
| --- | --- | --- | --- | --- |
| Arabidopsis thaliana | 14112 | 6259 | 165 | 4454 |
| Crataegus pinnatifida | 20697 | 16524 | 165 | 824 |
| Fragaria vesca | 13046 | 14258 | 165 | 3163 |
| Gillenia trifoliata | 12670 | 11739 | 165 | 1410 |
| Malus domestica | 19732 | 17348 | 165 | 537 |
| Malus sieversii | 19431 | 21457 | 165 | 1645 |
| Potentilla anserina | 23550 | 18278 | 165 | 1388 |
| Prunus armeniaca | 12291 | 14651 | 165 | 1879 |
| Prunus avium | 13603 | 17154 | 165 | 5745 |
| Pseudocydonia sinensis | 18542 | 16392 | 165 | 311 |
| Pyrus pyrifolia | 20641 | 20166 | 165 | 1498 |
| Rosa chinensis | 15173 | 18111 | 165 | 4367 |
| Rubus idaeus | 13238 | 17085 | 165 | 1160 |
| Rubus occidentalis | 12203 | 15168 | 165 | 1763 |

**Table 14.** Statistics of gene family clustering for the 14 species

|  | Genes | Ortho-logous gene | unassigned genes | Orthologous gene (%) | unassigned genes(%) | orthogroups containing species | orthogroups containing species(%) | species-specific orthogroups | species-specific gene | species-specific gene(%) |
| --- | --- | --- | --- | --- | --- | --- | --- | --- | --- | --- |
| Arabidopsis thaliana | 27287 | 24990 | 2297 | 91.6 | 8.4 | 14309 | 38.8 | 898 | 4454 | 16.3 |
| Pseudocydonia sinensis | 37742 | 35410 | 2332 | 93.8 | 6.2 | 18380 | 49.8 | 116 | 311 | 0.8 |
| Crataegus pinnatifida | 40568 | 38210 | 2358 | 94.2 | 5.8 | 18328 | 49.7 | 338 | 824 | 2 |
| Fragaria vesca | 33795 | 30632 | 3163 | 90.6 | 9.4 | 18249 | 49.4 | 805 | 3163 | 9.4 |
| Gillenia trifoliata | 28847 | 25984 | 2863 | 90.1 | 9.9 | 17848 | 48.4 | 468 | 1410 | 4.9 |
| Malus sieversii | 45143 | 42698 | 2445 | 94.6 | 5.4 | 19993 | 54.2 | 499 | 1645 | 3.6 |
| Malus dometica | 40548 | 37782 | 2766 | 93.2 | 6.8 | 19146 | 51.9 | 174 | 537 | 1.3 |
| Potentilla anserina | 46494 | 43381 | 3113 | 93.3 | 6.7 | 18136 | 49.1 | 601 | 1388 | 3 |
| Prunus armeniaca | 30432 | 28986 | 1446 | 95.2 | 4.8 | 16701 | 45.2 | 332 | 1879 | 6.2 |
| Prunus avium | 38273 | 36667 | 1606 | 95.8 | 4.2 | 17747 | 48.1 | 559 | 5745 | 15 |
| Pyrus pyrifolia | 44873 | 42470 | 2403 | 94.6 | 5.4 | 19145 | 51.9 | 397 | 1498 | 3.3 |
| Rosa chinensis | 44047 | 37816 | 6231 | 85.9 | 14.1 | 19907 | 53.9 | 1338 | 4367 | 9.9 |
| Rubus idaeus | 33857 | 31648 | 2209 | 93.5 | 6.5 | 20155 | 54.6 | 379 | 1160 | 3.4 |
| Rubus occidentalis | 32350 | 29299 | 3051 | 90.6 | 9.4 | 19245 | 52.1 | 564 | 1763 | 5.4 |


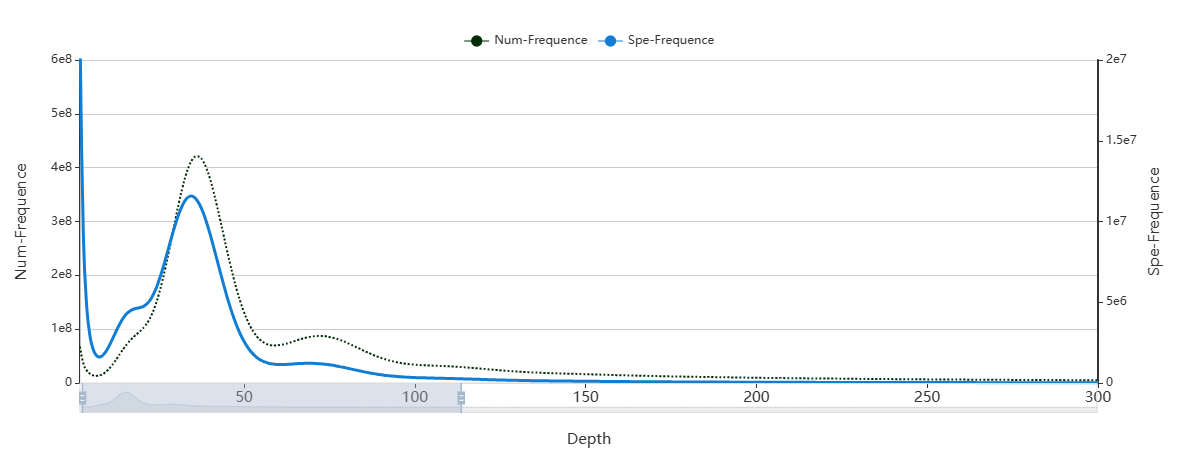


**Figure 1**.X-axis: "K-mer depth", Y-axis: "Number of K-mers corresponding to each depth".


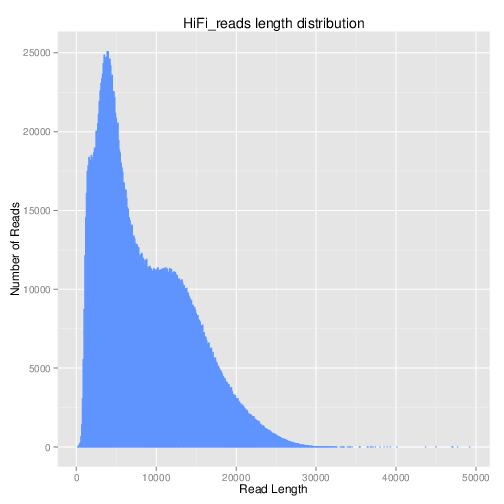


**Figure 2**. hifi data quality assessment chart


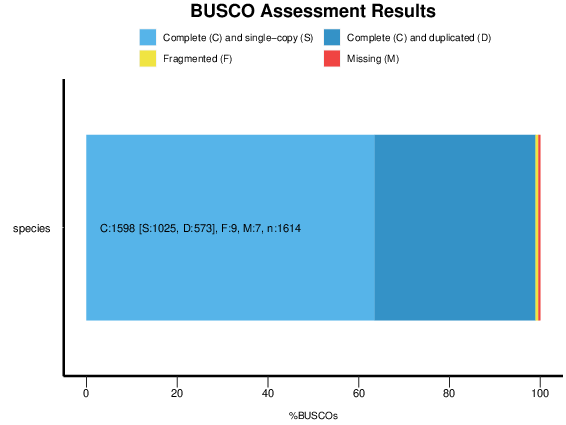


**Figure 3**. assessment results of BUSCOs. C：Complete BUSCOs, S：Complete and single-copy BUSCOs, D：Complete Duplicated BUSCOs, F：Fragmented BUSCOs, M：Missing BUSCOs, n：Total BUSCO groups searched.


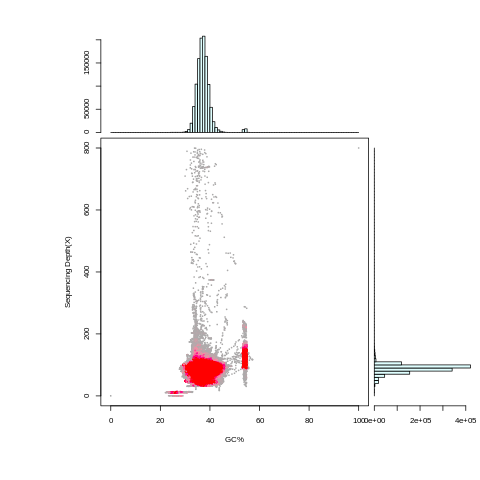


**Figure 4**. The GC content and average depth of the assembled genome sequence were calculated using 10k as windows and mapped to analyze whether there was GC bias in the sequencing data and whether there was contamination in the sample. GC content is concentrated around 37.32%, and there is no obvious separation in the scatter diagram, that is, no obvious separation of GC, indicating that there is no other exogenous pollution in the genome.


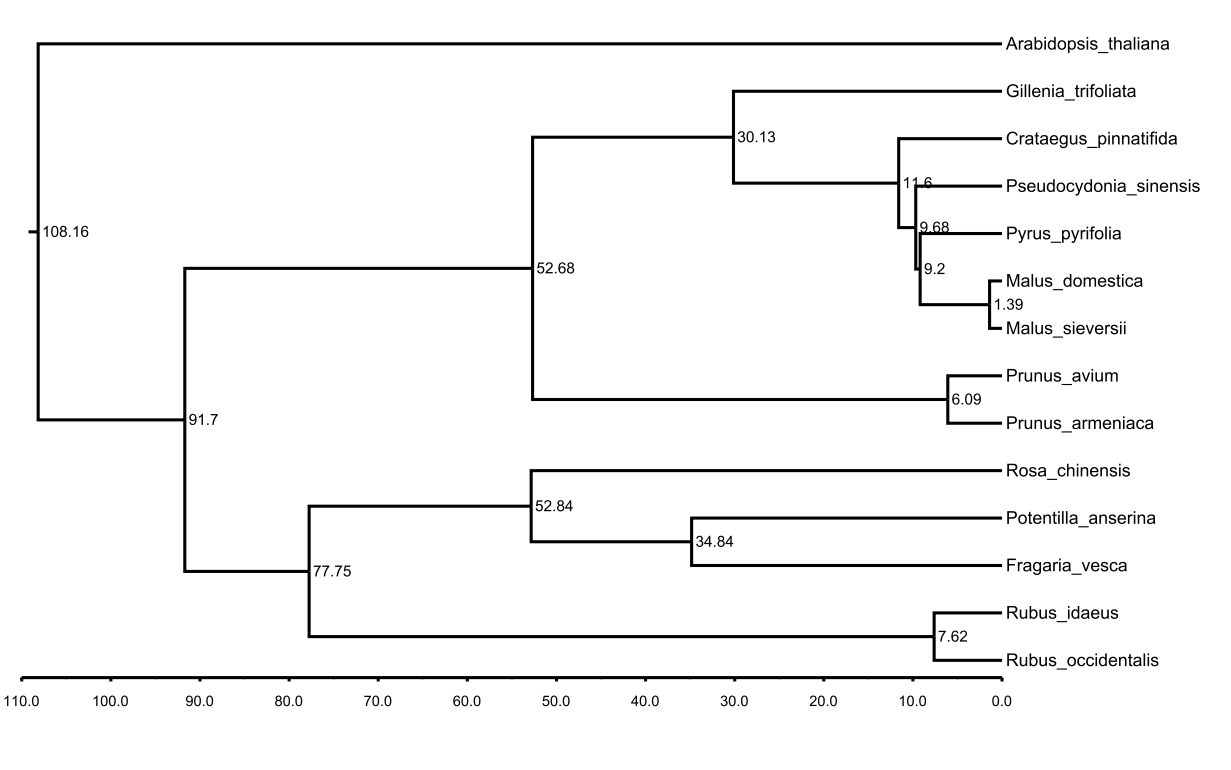


Figure 5. Divergent time trees of 14 species
